# Hydrogel-Core Microstructured Polymer Optical Fibers for Selective Fiber Enhanced Raman Spectroscopy

**DOI:** 10.3390/s21051845

**Published:** 2021-03-06

**Authors:** Mikel Azkune, Igor Ayesta, Leire Ruiz-Rubio, Eneko Arrospide, Jose Luis Vilas-Vilela, Joseba Zubia

**Affiliations:** 1 Department of Electronic Technology, Engineering School of Bilbao, University of the Basque Country (UPV/EHU), Torres Quevedo 1, 48013 Bilbao, Spain; 2 Department of Applied Mathematics, Engineering School of Bilbao, University of the Basque Country (UPV/EHU), Torres Quevedo 1, 48013 Bilbao, Spain; igor.ayesta@ehu.eus (I.A.); eneko.arrospide@ehu.eus (E.A.); 3 Macromolecular Chemistry Research Group (LQM), Department of Physical Chemistry, Faculty of Science and Technology, University of the Basque Country (UPV/EHU), Barrio Sarriena s/n, 48940 Leioa, Spain; leire.ruiz@ehu.eus (L.R.-R.); joseluis.vilas@ehu.eus (J.L.V.-V.); 4 BCMaterials, Basque Center for Materials, Applications and Nanostructures, UPV/EHU Science Park, 48940 Leioa, Spain; 5 Department of Communications Engineering, Engineering School of Bilbao, University of the Basque Country (UPV/EHU), Plaza Ingeniero Torres Quevedo, 1, 48013 Bilbao, Spain; joseba.zubia@ehu.eus

**Keywords:** Fiber Enhanced Raman Spectroscopy, microstructured Polymer Optical Fibers, liquid-core mPOF, hydrogel-core mPOF

## Abstract

A new approach of Fiber Enhanced Raman Spectroscopy (FERS) is described within this article based on the use of Hydrogel-Core microstructured Polymer Optical Fibers (HyC-mPOF). The incorporation of the hydrogel only on the core of the Hollow-Core microstructured Polymer Optical Fiber (HC-mPOF) enables to perform FERS measurements in a functionalized matrix, enabling high selectivity Raman measurements. The hydrogel formation was continuously monitored and quantified using a Principal Component Analysis verifying the coherence between the components and the Raman spectrum of the hydrogel. The performed measurements with high and low affinity target molecules prove the feasibility of the presented HyC-mPOF platform.

## 1. Introduction

New approaches of biosensors that join low-cost, high sensitivity and high specificity characteristics are widely pursued by the scientific community [1]. There is a wide range of application fields where the detection and quantification of different substances in low concentration are indispensable requirements, as well as clinical diagnosis, food safety, drug discovery or evaluation of hazardous contaminants, among others. Development of solutions based on polymer optical fibers (POF) in combination with highly functional matrixes, such as hydrogels, fit well with the aforementioned needs [2,3].

One of the most used spectroscopy techniques by means of biosensors is the Raman spectroscopy, due to its capability of providing the user with a deep molecular information about the sample. The inelastic dispersion suffered by the incident beam when it strikes the sample, describes the intramolecular information as well as the intermolecular interactions of the target [4,5]. This technique has been widely employed in different fields [6,7,8], such as the pharmaceutical drug monitoring [9] or food quality studies [10]. Additionally, the feasibility of this technique to combine with multivariate analysis based on Principal Component Analysis (PCA) has improved the boundaries of the technique [11,12].

However, due to its main drawback, i.e., the low efficiency of the Raman effect, many different approaches have been developed to enhance the dispersed signal. One of the most promising techniques is the Fiber-Enhanced Raman Spectroscopy (FERS), where the use of different microstructured optical fibers enhance the Raman signal. This improvement is based on the guidance of the scattered light and a much higher interaction between light and sample, as compared to the traditional Raman measurements [13]. Historically, silica microstructured optical fibers or also called Photonic Crystal Fibers (PCF) made by silica have been the most ones used [6,14,15,16]. Taking into account the most constraining requirement for this type of sensing platform—that is, the low cost—the use of microstructured Polymer Optical Fibers (mPOF) is a good alternative in comparison to the silica counterparts which have been employed in other works [17,18,19]. In order to be able to measure FERS spectra, the employed mPOFs need to be hollow-core mPOFs (HC-mPOF) which will be lately filled selectively with the solution of interest, creating liquid-core mPOFs (LC-mPOF), and therefore ensure the guidance of the incident and scattered lights. This technique has been already employed for glucose monitoring purposes [20]. However, in order to employ LC-mPOFs in complex solutions, functionalized matrixes, such as hydrogels, could be added to their core, which selectively discriminate between desirable and non-desirable molecules. This fact makes the platform more feasible and opens up a wide range of application possibilities [21].

More concretely, hydrogels present a high affinity for metal cations, dyes and many other molecules, being successfully employed as superabsorbent materials for the remediation of contaminants from water [22,23,24,25,26]. Heavy metals present in water could be responsible for many health issues in human beings and aquatic organisms directly or indirectly when coming in contact [27]. For example, nickel could cause lung embolism, heart disorders, respiratory failure or birth defects, among other health problems [28]. Evaluating the presence of metals in water is a key factor to select an adequate remediation strategy to effectively recover polluted water [29] or to prevent future health issues in humans. Among the possible hydrogels capable to efficiently remove heavy metals from the water, anionic hydrogels, such as poly(acrylic acid) [30], poly(methacrylic acid) [31] or sodium alginate [32], present the highest affinity due to their capability to coordinative interaction between metal and carboxylic acids present in these polymers. In this work, sodium alginate was used for hydrogel formation. It is important to notice that the monomer must fill the hollow core in the first instance in order to form the hydrogel in the hollow core of the fiber, and then, the crosslinking process must be carried out. There are several options available for this kind of approach. However, the most common crosslinking processes, usually by thermal or photopolymerization, are not adequate in this case since the temperature requirement in the first case and the UV light in the second case could produce undesired variation on the POF. In order to overcome these drawbacks, sodium alginate is a highly suitable choice due to its crosslinking capability with calcium cations that forms a stable Ca-alginate hydrogel [33], allowing its complexation once the hollow core of the fiber is filled.

This article reports a novel FERS platform based on the use of Hydrogel-Core microstructured Polymer Optical Fibers (HyC-mPOF). First, all the materials and methods are explained, starting from the fabrication of the probe and following with the later data processing. Afterwards, experimental measurements of the creation of the hydrogel within the core of the HC-mPOF and their use for FERS measurements are displayed and their discussion developed. Finally, the main conclusions obtained from the previous sections are summarized in the last section.

## 2. Materials and Methods

### 2.1. HC-mPOF Fabrication and Modification

**Figure 1:**
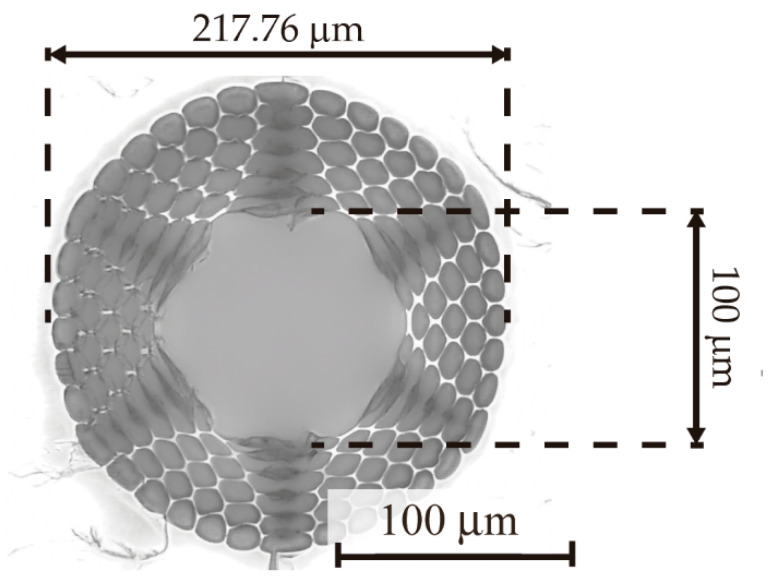
Cross-section microscope photograph of the HC-mPOF (microstructure. The central diameter of the microstructure is 100 µm, the diameter of the cladding 217.76 µm and the external diameter of the fiber is 1 mm.

The fabrication process of the HC-mPOFs employed in this work is well divided in three different steps. The first one corresponds to the fabrication of the fiber preforms, which were done using a computer numerical control machine for drilling 60-mm-wide poly(methyl methacrylate) (PMMA) rods. Afterwards, an annealing process was carried out in order to remove all moisture vestige. Then, the preforms were ready for the second fabrication step as it was drawn to the pursued diameter. During this process, a PMMA cane was added to the fibers to reinforce them and to protect the microstructure. The chosen microstructure followed a 6-ring pattern surrounding a central hollow-core, as can be observed in Figure 1. The central diameter of the mPOFs was 100 μm and the external diameter was 1 mm, with a core-cladding ratio of 0.47. These two-fabrication processes are further explained in our previous work [20].

Finally, in the third step, the fabricated HC-mPOFs were cut in 10-cm-length probes. Then, one of the end-faces of the probes were modified in order to fill selectively the core by means of capillary effect. With the aim of collapsing the cladding holes, but leaving open the central hollow-core, a widely reported method was carried out [34,35]. One of the two unmodified end-faces were immersed for two seconds in a 1/4 *v*/*v* NOA 65 in isopropyl alcohol solution and cured using a UV lamp afterwards. At this stage, due to the different capillary-filling speeds in the cladding holes and in the central core, the fiber probes were cleaved in the section where the core was still collapsed but the cladding holes remained opened. By immersing again these end-faces in the same solution for 10 s, the solution reached more height in the cladding holes than the remaining cured material in the core. Therefore, cleaving them between the two heights, the pursued modified end-faces were obtained, i.e., with the central core opened but the cladding holes sealed. Thus, the modified end-faces were the lower ones that would be immersed in different solutions and the unmodified end-faces were the upper ones that would be focused with the Raman microscope. In all cases, the final length of the fiber probes was 5 cm.

### 2.2. Alginate Hydrogel Formation in the Core of the mPOF

Sodium alginate (SA) powder and calcium chloride (CaCl_2_), used as crosslinking agent, were purchased from Sigma Aldrich (St Louis, MO, USA) and Panreac (Madrid, Spain), respectively.

**Figure 2:**
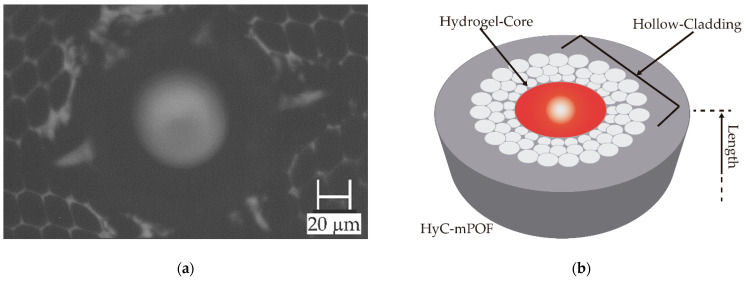
(**a**) Cross-section microscope photograph of a HyC-mPOF end-face. The presence of the hydrogel is observed as the blurred circle on the hollow of the microstructure. A HyC-mPOF end-face image obtained from the microscope; (**b**) a 3D illustration of the upper end-face of a HyC-mPOF.

In order to fill the hollow core of the HC-mPOFs, the surrounding pores were previously blocked. Then, the 5-cm-long fiber probes were immersed on 1% wt SA in aqueous solution, and left to fill the core by capillarity. Once the cores were filled, the fibers were dried, to improve the subsequent absorption of the Ca^2+^ solution (0.5 g/L), which acted as a crosslinker. The swelling process of the alginate eased the diffusion of the cations through all the area and increased the homogeneity of the formed hydrogel. Each step of the process was monitored by Raman microscopy. A microscope image of the upper end-face once the hydrogel was created is shown in Figure 2a. Additionally, an illustration of the upper end-face has been added for the sake of clarity in Figure 2b.

### 2.3. Experimental Set-Up

All the Raman spectra were recorded employing the same experimental set‑up, which consisted of ad-hoc fiber holder on a motorized xyz stage. This set-up enabled the accurate focus on the upper (unmodified) end-face, as well as the correct immersion of the lower (modified) end-face in different solutions. Depending on the process and the solution properties, there was a high variability on the time the solution needed to arrive at the upper end-face towards capillary effect. However, as all the measured processes were measured for several hours and using fiber probes with the same length, this rising time was negligible.

The Raman equipment employed for performing the Raman measurements was a Renishaw (Gloucestershire, UK) inVia confocal Raman Microscope. The excitation wavelength of the laser was 785 nm and the power launched to the sample was of 27.61 mW for 10 s each measurement. With this power and time exposition, any thermal effect on the sample or fiber-probe was discarded. The spectra were recorded from 100 cm^−1^ to 3200 cm^−1^ using a 20 X objective with a 0.4 NA from Leica (Wetzlar, Germany).

### 2.4. Data Processing

All the spectra recorded in different measurements were processed in first instance with a script developed in R software. First, the recorded spectra employing a hyperspec object [36], were smoothed implementing a Savitzky-Golay filter, and then, the baselines were removed using the 4S Fill Peaks algorithm [37,38].

For the peak tracking, different single wavenumbers were taken as the representative peak for each target molecule. On the one hand, the chosen peak for the nickel nitrate was the one placed at 1050 cm^−1^, which is extensively used in bibliography [39] and it corresponds to the *v*_3_(NO_3_^−^) band. On the other hand, the band placed at 2140 cm^−1^ was employed for the case of potassium ferricyanide (K_3_[Fe(CN)_6_]), whose unique peak placed at this wavenumber is commonly used for peak tracking. In order to normalize their intensity with an internal standard, they were normalized with a well-known PMMA reference peak placed at 810 cm^−1^ as this material is the subtract material and appears significantly in the FERS measurements.

The PCA models were created by a specifically developed commercial software called SIMCA (Sartorious Stedim Data Analytics AB, Umeå, Sweden). This software was basically employed for determining the state of the hydrogel formation as the tracking of a single peak for this aim was not enough.

## 3. Results

The results are divided in two different sections. The first measurements were focused on the creation of the hydrogel within the core of the fibers, to obtain HyC-mPOF probes. Afterwards, these HyC-mPOFs were tested for measuring whether high affinity or low affinity target molecules in order to verify their feasibility as sensing platform.

### 3.1. HyC-mPOF Creation and Hydrogel Creation State Quantification

As a way to confirm the creation of the hydrogel, and therefore, the creation of a HyC-mPOF probe, several measurements were performed following the next steps: Firstly, a Raman measurement focusing the hollow-core of the HC-mPOF probes was recorded to ensure the correct state of the fiber. Secondly, the lower end-face of the probes was immersed in a 1% wt alginate in deionized H_2_O solution, and another Raman spectrum was recorded. Finally, the lower end-face of the probes were removed from the alginate and immersed in a 0.5 *w*/*v* (g/L) calcium solution for three hours and a Raman measurement was taken every five minutes. The progression of the obtained spectra showed the creation of the hydrogel within the core; besides, they were used for creating a PCA model to verify the state of the creation and therefore, to classify the probes.

#### 3.1.1. Raman Spectra Progression

**Figure 3:**
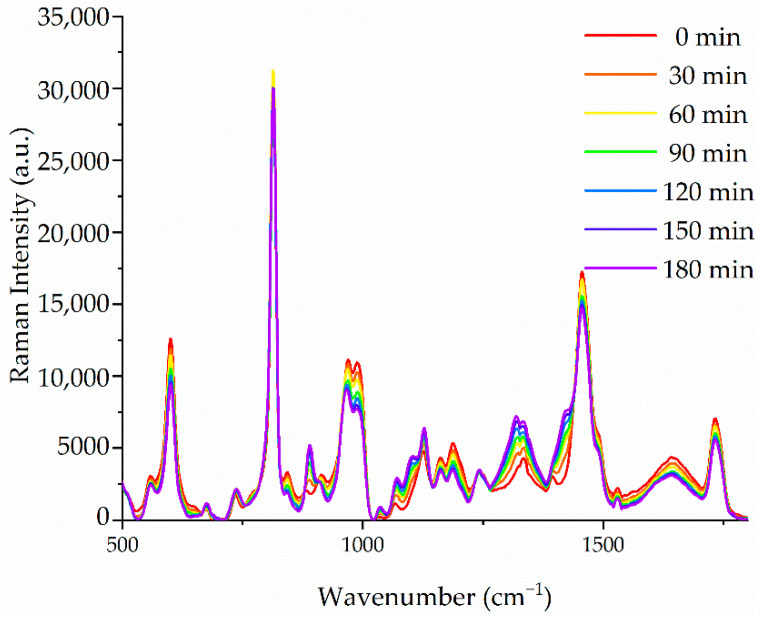
Raman spectra progression of the hydrogel formation for an acquisition period of 3 h.

The measured progression of the hydrogel creation is plotted in Figure 3. Regarding these spectra, background peaks corresponding to PMMA appear in first instance. These spectra modify as the immersion time increases and peaks corresponding with the hydrogel tend to prevail.

As it can be noted regarding the spectra of Figure 3, many peaks alter the initial PMMA background signal. This means that the tracking of a single peak is not a correct method to guarantee the characterization of the hydrogel formation. As a way to overcome this issue, all the spectra recorded in this step were the input to create a PCA model and obtain principal components able to describe an accurate state of the hydrogel creation.

#### 3.1.2. PCA Model of the Hydrogel Creation

**Figure 4:**
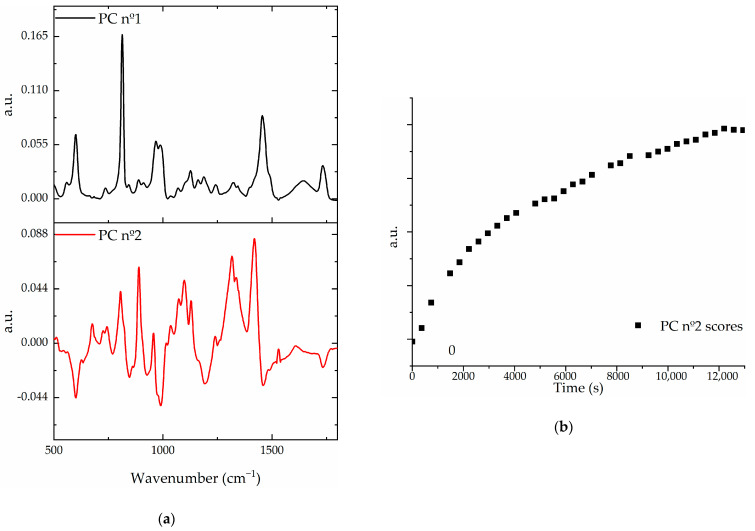
(**a**) The spectral shapes of the first and the second principal components (PC nº1 and nº2, respectively) of the PCA model that describes the hydrogel creation; (**b**) The temporal evolution of the scores corresponding to the PC nº2.

The PCA model consisted of 180 spectra corresponding to five different measurements joined to create the database. Each measurement consisted of 36 spectra recorded every 5 min once the lower end-faces were immersed in the alginate solution and kept them for three hours. The first two components of the built PCA model and a score progression of the second component of an example HyC-mPOF creation are shown in Figure 4a,b, respectively:

Analyzing the components of the model, it could be seen that the second one (red) fits accurately with the Raman spectrum of the hydrogel [40], since the main prominent peaks of this component fits with the main peaks of the Ca-alginate, such as the glycosidic ring breathing mode located at 1088 cm^−1^ or the symmetric carboxylate stretching vibration placed at 1433 cm^−1^. Measuring the scores of the component, for different measurements, their values are increased chronologically as it is shown in Figure 4b. Therefore, it may be concluded that regarding this second component, an accurate state of the hydrogel formation is obtained, in the sense that if the score of a HyC-mPOF probe follows this pattern, it is considered a correct probe for forthcoming measurements.

### 3.2. High and Low Affinity Molecule Detection

The next step for proving the feasibility of the HyC-mPOFs as selective sensors was to test them with different solutions, containing high affinity molecules, nickel nitrate, or low affinity ones, potassium ferricyanide. Once the probes had the hydrogel created within their cores, the lower end-faces of the probes were immersed in different solutions for several hours and Raman spectra was recorded every 5 min.

#### 3.2.1. High Affinity Molecule Detection

**Figure 5:**
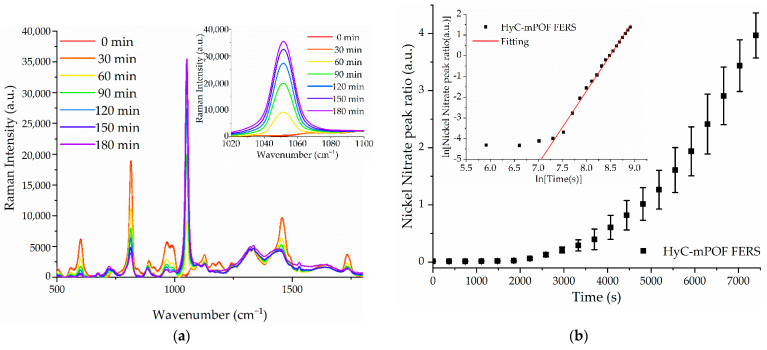
(**a**) Raman spectra progression during 3 h in a nickel nitrate detection measurement. The inset shows the spectral evolution around 1050 cm^−1^ in more detail; (**b**) The average values together with their corresponding error bars of the nickel nitrate peak ratio of different measurements. The inset shows the mean value of nickel nitrate peak ratio measurements for a log-log chart and the red line corresponds to its linear fitting with a slope of 3.5 (coefficients of determination of R^2^ = 0.994).

Nickel nitrate has a prominent peak placed around 1050 cm^−1^, so by tracking it during the immersion time, the concentration of the solution can be determined. All the measured probes were immersed in a solution containing 0.1 mol/L nickel nitrate in H_2_O. In Figure 5a, a Raman spectra progression is shown, together with the ratio of the main nickel nitrate peak (at 1050 cm^−1^) normalized with the main PMMA peak (at 810 cm^−1^) in Figure 5b.

Figure 5a describes how the HyC-mPOF sensor is able to detect the target molecule as the peak located in 1050 cm^−1^ arises substantially. In order to parse these results, in Figure 5b, the average of peak ratios of three different measurements are shown. In this last graph, a clear exponential tendency is observed. This behavior can be explained as follows: on the one hand, during the first seconds of the experiments, the swelling of the hydrogel takes place, in which the detection capacity is still low. As the hydrogel is completely swell, around 1500 s afterwards, the nickel nitrate molecules are diffused through it more easily and can be coordinated with the carboxylic group, increasing the acquired Raman signal. Moreover, the target molecules also need a certain time to reach the Raman active length of the probe, some few centimeters. The five-centimeter length of the probes is the minimum for the correct manipulation of the probes, so the time that passes without any detection is acceptable considering the overall benefits.

**Figure 6:**
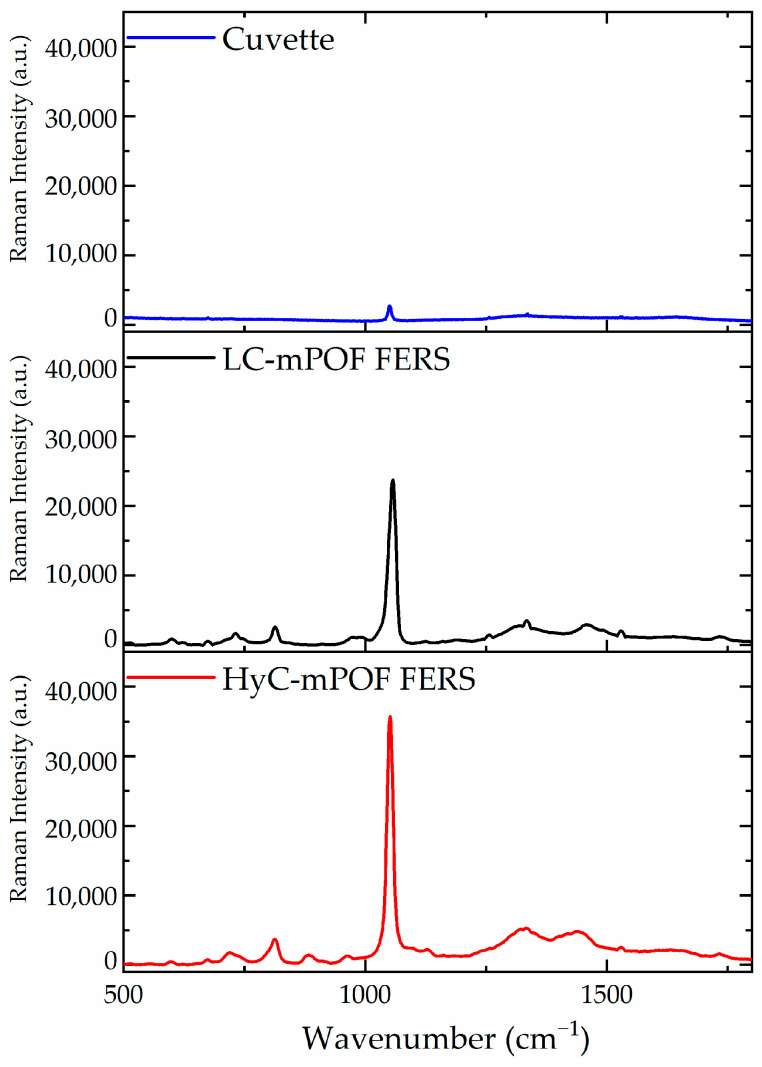
Comparison stack among cuvette (blue), LC-mPOF (liquid-core mPOF) (black) and HyC-mPOF (red) Raman measurements. In all cases, the Raman peak of the nickel nitrate is observed at 1050 cm^−1^ but with different intensities.

Besides, the obtained measurements were also compared to a simple solution measurement in a cuvette and a LC-mPOF measurement. The obtained results are shown in Figure 6.

Comparing the HyC-mPOF acquisition to the cuvette measurement, a strong enhancement of the nickel nitrate peak detection appears by means of FERS, showing the advantages of the technique. Additionally, the difference between LC-mPOF and HyC-mPOF is not very noticeable, as the basis of the techniques are the same and the light transmission is similar through the core of the probes filled by liquid or hydrogel. However, the selectivity offered by the presented platform makes this technique favorable for high affinity molecule detection.

#### 3.2.2. Low Affinity Molecule Detection

**Figure 7:**
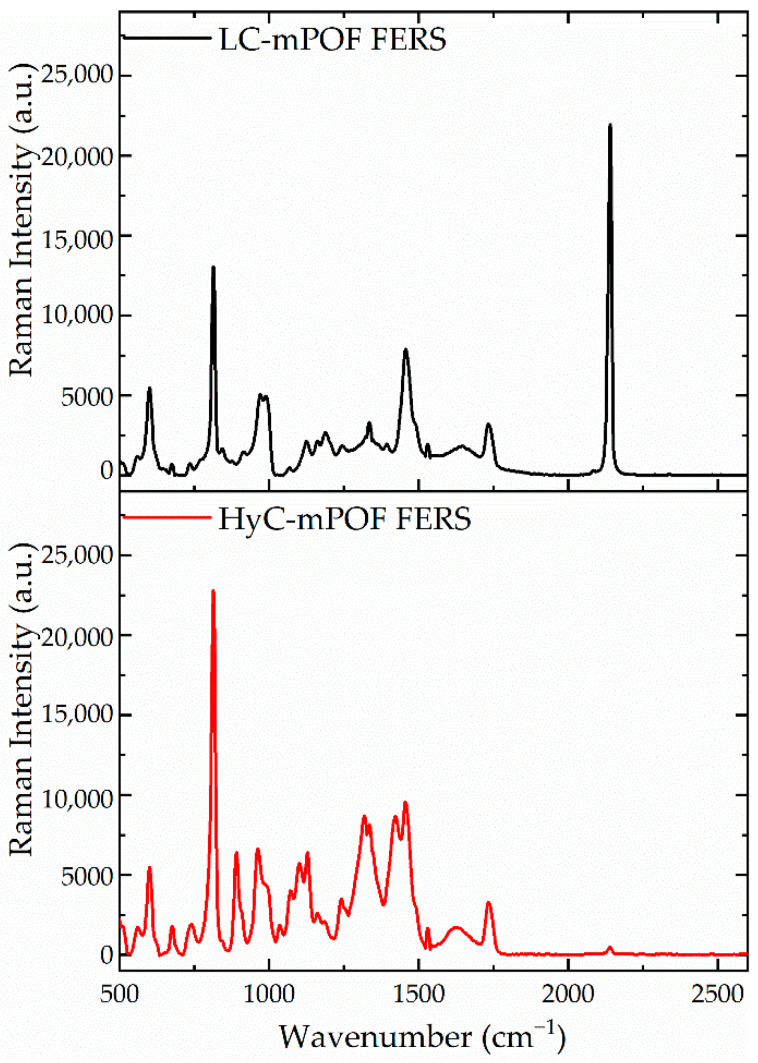
Comparison of two Raman measurements employing a LC-mPOF (black) and a HyC-mPOF (red) during six hours of immersion. In both cases, the Raman peak of the potassium ferricyanide is observed at 2140 cm^−1^ but with different intensities.

HyC-mPOFs were then tested for low affinity molecules, in this case, potassium ferricyanide. This target molecules show low affinity since the monovalent cations, K^+^, do not coordinate with –COOH groups of the alginate, and the [Fe(CN)_6_]^4−^ is a highly voluminous anion that is repelled by the deprotonated carboxylic groups of the alginate present at water pH (pH = 6). The performed measurements were replicated from the previous sections, the lower end-faces of the probes were immersed in a 0.1 mol/L potassium ferricyanide solution in H_2_O for several hours and their Raman spectra recorded. The obtained results are shown in Figure 7.

If the recorded spectra are compared, after the probes are immersed for 6 h in the solution, it could be noted that LC-mPOF shows a high peak nearby 2140 cm^−1^, where the potassium ferricyanide has its unique peak, whereas in the HyC-mPOF, an extremely weak peak appears in this wavenumber. That means that the HyC-mPOF does not let the potassium ferricyanide to access the Raman active volume of the HyC-mPOF through the hydrogel, and therefore, it shows the already mentioned and desired selectivity of the presented platform. Various measurements were carried out, three for each platform in order to assure the phenomena.

## 4. Conclusions

A new FERS platform has been described by creating a functional hydrogel selectively within the core of an ad-hoc fabricated HC-mPOF. The fabrication and modifications suffered by the HC-mPOF have been described, as well as the creation of the hydrogel and its monitorization due to a PCA model. The reliability of the second component in order to quantify the hydrogel creation process has been proved. Afterwards, the sensing platform has been demonstrated for high and low affinity molecules, nickel nitrate and potassium ferricyanide respectively. Results show that the implemented HyC-mPOF is able to detect nickel nitrate, whereas the potassium ferricyanide keeps undetectable even for six hours of measurement. The selective behavior of the platform offers several advantages compared to cuvette or to LC-mPOF measurements. Moreover, the presented platform shows high potential for further development in which high selectivity and specificity but low-cost platforms are required.

## Data Availability

Not applicable.
